# Association of GST gene polymorphism and noise-induced hearing loss: GST gene polymorphism and NIHL

**DOI:** 10.3934/publichealth.2019.4.546

**Published:** 2019-12-11

**Authors:** Ziba Loukzadeh, Hadi Eshaghi Sani, Mohammad Hasan Sheikhha, Farzaneh Morteza Ratki

**Affiliations:** 1 Associate professor, Industrial Diseases Research Center, Center of Excellence for Occupational Medicine, Shahid Sadoughi University of Medical Sciences, Yazd, Iran; 2 Assistant professor, Department of Occupational Medicine, Faculty of Medicine, Hormozgan University of Medical Sciences, Bandar Abbas, Iran; 3 Professor of Medical Genetics, Stem Cell Biology Research Center, Yazd Reproductive Sciences Institute, Shahid Sadoughi University of Medical Sciences, Yazd, Iran; 4 Department of Biology, Faculty of Science, Yazd University, Yazd, Iran

**Keywords:** noise, noise induced hearing loss, polymorphism, GSTT1, GSTM1

## Abstract

**Background:**

It has been proposed that Noise-induced hearing loss is a complex disease that is combination of environmental and genetic factors. There are inconsistent results concerning the association between variation in glutathione S-transferase (*GST*) genetic polymorphisms (*GSTT1* rs1049055 and *GSTM1* rs10712361) and susceptibility to Noise-induced hearing loss.

**Objective:**

This study was designed to assess the association between *GST* gene polymorphism and Noise-induced hearing loss among noise-exposed workers. *Methods:* In a case-control study, male workers from tile and ceramic factories were selected randomly. Subjects were classified into two groups according to the result of audiometry: 73 subjects showed Noise-induced hearing loss which was considered in the case group and 87 subjects without hearing loss was enrolled in the control group. The *GSTT1* and *GSTM1* polymorphism of both groups were assessed by multiplex polymerase chain reaction.

**Results:**

Null *GSTT1* and *GSTM1* genotypes were more frequent in case group but no significant statistical difference was seen in case and control groups. No significant link between *GSTT1* and *GSTM1* genotypes was found.

**Conclusion:**

This study suggests that the genetic variability of *GSTT1* and *GSTM1* has no effect on susceptibility to noise induced hearing loss.

## 1. Introduction

Noise-induced hearing loss (NIHL) is one of the most common occupational disease experienced across the world in occupational environments [1] and the second most common cause of acquired hearing loss after presbyacusis [2]. It has been estimated that about 500 million individuals might be at increased risk of developing NIHL [3].

There is a large individual variability in susceptibility to the damaging effects of noise; after exposure to similar level of noise, some subjects develop different level of NIHL, so has been proposed that NIHL is a complex disease that is influenced by interaction of environmental and genetic factors [4]. Environmental risk factors causing hearing loss include noise, vibration, heat, chemicals such as organic solvents and heavy metals, ototoxic drugs, infections, nutrient disturbances, smoking, high blood pressure and cholesterol levels and possibly pigmentation [4],[5].

Little is known about the involvement of genetic factors that influence NIHL in human. Several NIHL susceptibility genes have been suggested, such as, genes encoding for the antioxidant systems, potassium ion channels, heat shock proteins, and the signaling function of the immune system [6]. Oxidative stress genes, such as glutathione S-transferase, (GST); glutathione peroxidase (GPX1); glutathione reductase (GSR), superoxide dismutases (SOD), catalase (CAT), and paraoxonase (PON) [6] are obvious candidate genes for NIHL [7]. Numerous studies have shown that high-intensity noise exposure causes mechanical or metabolic changes in the cochlea which lead inner ear damage [8]. The metabolic changes are caused mainly by increase in free radical species (FRS) that play a primary role in NIHL [9]. The harmful effects of FRS are generally prevented by endogenous antioxidants. Tripeptide glutathione (GSH), a major intracellular antioxidant, is proven to detoxify free radicals and to prevent NIHL [10]. The most important antioxidant enzymes in the cochlea that are involved in GSH metabolism are glutathione S-transferase (GST), glutathione peroxidase (GPX1), and glutathione reductase (GSR) [10]. GST enzymes catalyze conjugation of GSH with free radicals and have an important role protecting the cochlea [11]. GST classified into several gene classes (GSTA, GSTM, GSTP, GSTT, GSTZ, GSTS, GSTK, GSTO) [5]. In humans, there is genetic variability of GSTT1 and GSTM1. Up to 50% of the Caucasian population has null genotype for GSTM1 [12], while 25–40% of the Caucasian population are null for GSTT1 gene [13]. Individuals with the null genotype for either GSTT1 or GSTM1 cannot conjugate toxins specific to these enzymes, so they may be at increased risk of developing NIHL [14].

There are limited and inconsistent results about association between variation in GST genes and susceptibility to NIHL. Rabinowitz et al. showed that among 58 noise-exposed American workers, GSTM1 null individuals had lower amplitudes of high frequency otoacustic emissions compared to individuals possessing the gene. However their finding lacked sufficient power and this may be a spurious finding [14]. In later study involving 194 Chinese workers who exposed to occupational noise, Yang et al. suggested that GSTT1 null individuals might be more susceptible to NIHL [15]. Lin et al. found that among 58 noise-exposed workers in Taiwan, individuals carrying all genotypes with GSTM1 null, GSTT1 null, and GSTP1 I1e^105^/I1e^105^ might be more susceptible to the effects of noise [10]. However in much more comprehensive study among 10% most susceptible and 10% most resistant to noise subjects selected from 1200 Swedish workers, an association between GSTT1, GSTM1, and GSTP1 could not be confirmed [11]. Palodetto et al. found no significant association between GST gene variants (GSTT1 and GSTM1) and ototoxicity of aminoglycosides, which seems to involve similar oxidative stress mechanisms [16]. In addition to contradictory results in various studies, the effect of genetic factors on susceptibility of NIHL can only be regarded as valid when they have been replicated in an independent population [17]; so, the present study investigates the association between the deletion polymorphism in the GSTT1 (rs1049055) and GSTM1 (rs10712361) genes and susceptibility to NIHL in Iranian population.

## 2. Materials and methods

This case-control study was performed in accordance with the guidelines in the Declaration of Helsinki and with approval from the medical ethics committee of Shahid Sadoughi University of medical sciences, Iran. An informed consent was obtained from all study subjects.

### 2.1. Subjects

The present study was conducted on male workers from 7 tile and ceramic factories in Yazd, a center province of Iran. Factories which had high stability of the workforce and had periodic noise level measurements were selected by simple random sampling from all tile and ceramic factories in Yazd (n = 30). In each factory subjects who had been employed for at least 3 years and exposed to hazardous noise (≥ 85 dBA) were selected by simple random sampling [11]. Noise level was extracted from the result of measurements routinely performed in the factories by industrial hygiene incorporations.

Those with previous history of acoustic trauma, congenital or familial hearing loss, and ototoxic drug consumption, positive medical history of diabetes mellitus, hypertension, head trauma, head surgery, chronic otitis media, conductive hearing loss in pure-tone audiometry, previous exposure to organic solvents or loud noise in previous or second job or in non-occupational form, and family history of deafness were excluded from the study.

A questionnaire including age, duration of employment in current job, smoking and alcohol consumption and medical history was filled for each subject.

After usual otoscopic examination, pure-tone audiometry (using clinical audiometer: Interacoustic, AC40, Denmark, headphone: TDH39) was performed for the subjects in an acoustic chamber meeting the requirements of the American National Standards Institution (ANSI) 2004 by an expert audiologist (blinded to the study) [12]. Air conduction was assessed at 250, 500, 1000, 2000, 3000, 4000, 6000, and 8000 Hz frequencies and bone conduction at 250–4000 Hz frequencies for each ear.

After considering inclusion and exclusion criteria, according to the result of audiometry, subjects were classified into two groups: from 80 NIHL subjects, 73 individuals with audiograms suggestive NIHL as a case group (8 were excluded due to diabetes mellitus, conductive hearing loss, familial hearing loss and history of head trauma) and from total 90 subjects, 87 normal hearing individuals enrolled in the study as controls [13], (3 were excluded due to conductive hearing loss and diabetes mellitus). Their workday was approximately 8 hours per day. Most workers did not use hearing protective devices (ear plugs) and a few workers used ear plugs very irregularly.

### 2.2. Molecular study

Venous blood samples for DNA extraction were also collected in EDTA tubes at the workplace and placed immediately container and then transported on dry ice to the laboratory. This extraction was carried out under sterile conditions and under the laminar hood. DNA extraction from blood was performed using a DNA extraction kit called Bio Flux (Shanghai Beiyi Bioequip Information Co., china). The next step was DNA extraction for use in the polymerase chain reaction. The purpose of the multiplex PCR was to amplify the genomic DNA regions containing GSTT1 (Forward primer: 5′ TTCCTTACTGGTCCTCACATCTC 3, Reverse primer: 5′ TCACCGGATCATGGCCAGCA 3′), and GSTM1 (Forward primer: 5′ GAACTCCCTGAAAAGCTAAAGC 3′, Reverse primer: 5′ GTTGGGCTCAAATATACGGTGG 3′) in addition to the B-globin gene as control. At this stage, 15 µl Master Mix 2x (cinnagen, Iran) (which itself contains 2mM MgCl2), was made up of a total volume of 30 µl: containing 100 ng of genomic DNA and 12.5 pM each of the forward and reverse GSTT1, GSTM1, and B-globin primers.

The PCR reaction was carried out in 3 steps, one cycle of 94 °C for 5 minutes followed by 30 cycles of 94 °C for 1 min, 62 °C for 1 min, and 72 °C for 1 min and the final step was 72 degrees for 5 minutes. The PCR product was run in 2% (w/v) agarose gel and the bands were visualized with UV light.

Three bands should be observed: The B-globin band to be observed in all wells is 268 bp, GSTT band is 480 bp and GSTM band is 219 bp. Both GSTM and GSTT bands may exist or have been deleted, creating four different patterns: T+ M+, T+ M−, T− M+ or T− M−.

### 2.3. Statistical analysis

Data was analyzed by SPSS version 18 (SPSS, Inc., Chicago, IL). One-Sample Kolmogorov-Smirnov test was applied to test normality of study variables. Independent Student's *t*-test and Mann-Whitney test were used to compare continuous variables between groups. The frequency of polymorphism was compared between groups by chi-square test and Fisher's exact test and odds ratios with corresponding 95% confidence intervals (95% CI) were calculated with Mantel-Haenszel method. *P*-value ≤ 0.05 was considered statistically significant.

## 3. Results

A total of 160 male subjects that comprised 73 individuals with audiograms suggestive NIHL as a case group and 87 normal hearing individuals as a controls participated in this study. Table 1 presents the demographic and personal characteristics of the study subjects.

**Table 1.** Characteristic of the study population.

	Case group (n = 73)	Control group (n = 87)	P-Value
Age (years)* (Mean±SD)	35.12 ± 3.85	34.71 ± 4.21	0.07
Employment (years)^†^ (Mean±SD)	6.40 ± 2.39	6.37 ± 1.72	0.90
Noise exposure (TWA) dBA^†^ (Mean±SD)	90.14 ± 2.59	89.72 ± 2.32	0.26
Smoking^‡^ 9N (%)]	19 (26.00%)	14 (16.10%)	0.09

Note: *Student's *t*-test; ^†^Mann-Whitney test; ^‡^Chi-square test.

Null GSTT1 and null GSTM1 were found in 33 persons (20.6%) and 54 individuals (33.8%) of the study population, respectively. The frequency of null GSTT1 and GSTM1 genotypes and interaction between these genes as well as the comparison of the results among NIHL and control groups are shown on table 2. Null GSTT1 and GSTM1 genotypes were more frequent in NIHL group but no significant statistical difference was seen in NIHL and control groups.

**Table 2.** Null genotypes from genes GSTT1 and GSTM1 in case and control groups.

Genotypes	Case group n (%)	Control group n (%)	p-value	0R (95% CI)
GSTT1				
Normal genotype	57 (78.1)	70 (80.5)	0.43	1.08 (0.72–1.61)
Null genotype	16 (21.9)	17 (19.5)		
GSTM1				
Normal genotype	45 (61.6)	61 (70.1)	0.17	1.22 (0.87–1.71)
Null genotype	28 (38.4)	26 (29.9)		

**Figure 1.**
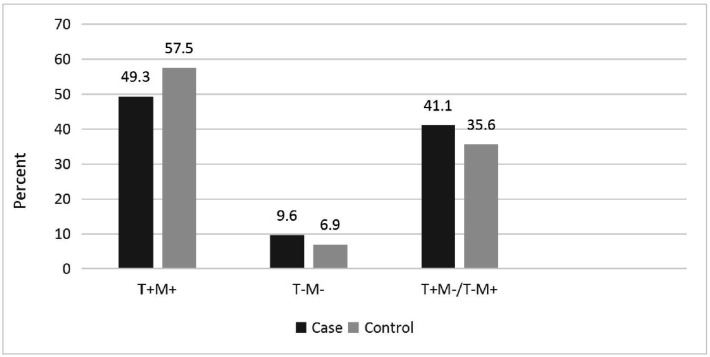
Interaction between GSTT1 and GSTM1 genotypes in case and control groups.

No significant interaction between GSTT1 and GSTM1 genotypes was found (P-Value > 0.05) (Figure 1).

## 4 Discussion

The aim of this study was to assess that genetic variability of GSTT1 and GSTM1 is associated with susceptibility to NIHL in Iranian population.

It seems that many factors play a role in NIHL including genetic and environmental factors. Subjects who developing NIHL after exposure to noise are often exposed to many other risk factors that interact with each other and/or with genetic factors [14].

This study did not detect that the genetic variability of GSTT1 and GSTM1 affect on susceptibility to noise induced hearing loss. There are many controversial studies on association variations in oxidative stress genes and susceptibility to NIHL. Carlsson et al. analyzed genetic polymorphism in the 10% most susceptible and 10% most resistant extremes of 1200 Swedish noise-exposed workers to investigate whether genetic variation is associated with susceptibility to NIHL. In their study no significant differences were found between 103 noise-susceptible and 114 noise-resistant Swedish workers from the point of view of GSTM1 and GSTT1 polymorphism [6]. Palodetto et al. showed that polymorphism in GSTM1 and GSTT1 have no influence on ototoxicity of aminoglycosides, which seem to involve similar oxidative stress mechanis like NIHL [14]. Rabinowitz et al. analyzed polymorphism status for GSTM1 and GSTT1 in 58 factory workers [9]. They found that carriers of GSTM1 gene had better high frequency otoacoustic emission (OAEs) compared to GSTM1 null individuals but they found no significant effect of GSTM1 with pure tone audiogram. On the other hand GSTT1 did not show a protective effect. Their study lacks sufficient power due to small sample size. On the other hand, in a study in China, there was no significant relationship between GSTM1 gene deletion NIHL but the results suggested that GSTT1 polymorphism might play an important role in the development of NIHL in Chinese workers and the individuals with the GSTT1 null genotype might be more susceptible to NIHL [15] while in another study on Chinese workers, individuals with the GSTM1 null genotype had a statistically significantly increased risk of NIHL [22]. Study on 53 Taiwan male workers showed that GST polymorphism may modify the susceptibility to NIHL and that individuals carrying all genotypes with GSTT1 null, GSTM1 null, and GSTP1 Ile(105)/Ile(105) are more susceptible to NIHL [5].

The findings of study on Brazilian subjects (107 subjects with NIHL, 44 with other causes of hearing loss and 104 controls) suggest effects of GSTT1 and GSTM1 polymorphism on the risk of NIHL [2]. In study conducted by Manche and et al. on 220 subjects with presbycusis, gene polymorphisms of GSTT1/M1were found to contribute significant risk to presbycusis [23].

Several studies showed that antioxidant system is activated after noise exposure. It seems that noise exposure levels are important in activation of this system. So, it is possible in studies that did not find association of GST gene and NIHL, like our study, the noise exposure levels to which the majority of study workers have been exposed over the years did not reach the critical level needed to activate the antioxidant system, and therefore differences in their antioxidant systems are unlikely to show up in their findings. However, it is possible that difference exists when subgroups (noise exposure levels, noise exposure times) be evaluated separately [6].

Our results showed that the observed frequency of null GSTT1 and null GSTM1 were 20.6% and 33.8%, respectively. The 20.6% frequency of null GSTT1 is on the low end of the range of 19–58% reported in some Asian populations also the 33.8%, frequency of null GST M1 is of the range of 28–63% reported in some Asian populations [5]. 25 to 40% of the European population has GSTT1 deletions also GSTM1 gene deletions have been reported up to 50% of European population [3].

Our study has several limitations. First of all, it was a cross-sectional study, and may therefore distort the association due to the healthy worker effect, and does not allow demonstration of a causal relationship. Secondly, this study lacks sufficient power due to small sample size. NIHL has complex nature that is influenced by interaction of environmental and genetic factors [14]; so many other environmental risk factors can have a confusing effect on this relationship, such as potential influence of length of time that the subjects have been exposed to non-occupational noisy conditions, commitment to using hearing protection devices at workplace and etc., As well as other risk factors (e.g., obesity, diet) have not been analyzed. A study conducted in 2006, suggested that to get better results on studies on NIHL susceptibility genes, 10% of the subjects with the worst hearing thresholds as susceptible to NIHL workers and 10% of the subjects with the best hearing thresholds as resistant to noise workers should classify by international reference standard such as ISO 1999:1990 standard [24].

Due to the impact of ethnic differences on the gene level, the associations can only be valid when they have been replicated in an independent population, therefore, this study was conducted for the first time on the Iranian population.

## 5. Conclusion

We did not detect that the genetic variability of GSTT1 and GSTM1 effect on susceptibility to noise induced hearing loss in Iranian population. Further studies should include workers with different noise exposure patterns. Combination of genes variation with other risk factors should also be analyzed.

## Take-home message

(1). Noise-induced hearing loss is a complex disease affected by both genetic and environmental factors.

(2). Several Noise-induced hearing loss susceptibility genes has been suggested but oxidative stress genes are obvious candidate genes. One of important antioxidant enzymes in the cochlea are glutathione S-transferase (GST).

(3). This study investigates the association between GST gene polymorphism and noise induced hearing loss among Iranian noise-exposed workers.

(4). Further studies should include different population with different noise exposure patterns such as noise exposure levels, noise exposure times.
